# Diagnostic Value of Superparamagnetic Iron Oxide Nanoparticles as a Tracer for Sentinel Lymph Node Mapping in Early-Stage Cervical Cancer: The Preliminary Clinical Experience

**DOI:** 10.3390/jfb16060196

**Published:** 2025-05-26

**Authors:** Marcin A. Jedryka, Andrzej Czekanski, Marcin Kryszpin, Tymoteusz Poprawski, Krzysztof Grobelak, Piotr Lepka, Rafał Matkowski

**Affiliations:** 1 Department of Oncology, Faculty of Medicine, Wroclaw Medical University, 50-367 Wroclaw, Poland; andrzej.czekanski@umw.edu.pl (A.C.); piotr.lepka@umw.edu.pl (P.L.); rafal.matkowski@umw.edu.pl (R.M.); 2 Department of Oncologic Gynecology, Lower Silesian Oncology, Hematology and Pulmonology Center, 53-413 Wroclaw, Poland; marcin.kryszpin@dcopih.pl (M.K.); tymoteusz.poprawski@dcopih.pl (T.P.); krzysztof.grobelak@dcopih.pl (K.G.); 3 Breast Cancer Clinic, Lower Silesian Oncology, Hematology and Pulmonology Center, 53-413 Wroclaw, Poland

**Keywords:** sentinel lymph node, tracer, mapping, lymphadenectomy, cervical cancer, superparamagnetic iron oxide, nanoparticle

## Abstract

Sentinel lymph node (SLN) mapping has been investigated as part of surgical staging in women with early-stage cervical cancer (CC); however, pelvic lymphadenectomy (PLND) remains the standard of care. This study aimed to assess feasibility and safety of SLN detection using superparamagnetic iron oxide (SPIO) nanoparticles as a tracer in CC. Thirty CC patients presumed to be stage I were included in this study with SPIO administered intracervically as a tracer for SLN mapping using a magnetometer and followed by PLND. The endpoints of the study included the proportion of successful SLN detection, the average number of SLNs per patient, and the proportion of pathologically positive results per patient and per node. The diagnostic accuracy of SPIO nanoparticles for detection of metastatic SLNs was evaluated by Receiver Operating Characteristic (ROC) curve analysis, with the area under the ROC curve (AUC) used to demonstrate the studied method’s sensitivity. Safety endpoints were a summary of all reported adverse events. SLNs were detected in all cases, bilaterally in 27 patients (90%). The median number of SLNs per patient was 3.5. Four cases had metastatic SLNs. The general malignancy rate per patient was 13.3%, and per node, it was 0.8%. The malignancy detection rate of SLNs was 100% per patient and 80% per node. The AUC of 1.0 (*p* < 0.001) confirmed the diagnostic value of the SPIO technique for the detection of metastatic SLNs, with a sensitivity of 100%. No adverse events related to the SPIO administration were reported. SPIO nanoparticles, as a tracer for SLN mapping in early-stage CC patients, demonstrated satisfactory accuracy parameters and safety; however, these data need to be evaluated by further research.

## 1. Introduction

Cervical cancer (CC) is the fourth most common cancer in women considering both incidence and mortality, with estimated 660,000 new cases and 350,000 deaths worldwide in 2022 [1]. In 2024, more than 2225 women were diagnosed with CC in Poland and 1552 of them died from this malignancy, according to the National Cancer Registry [2]. The oncologic outcomes of CC management depend mainly on the cancer stage at diagnosis, with poor prognosis for high-stage patients but with excellent results foroverall survival rates (85–90%) for early-stage disease [3,4]. The early-stage CC population comprises stages IA1 with lymphovascular space involvement (LVSI), IA2, IB1, IB2, and IIA1, according to the International Federation of Gynecology and Obstetrics (FIGO) recent update [5]. Early CC spreads mostly via the lymphatic system; hence, the knowledge of lymph node (LN) status is the most important factor for survival and eventual postoperative adjuvant therapy [6,7]. Therefore, pelvic lymph node dissection (PLND) in these patients is a key surgical procedure along with radical hysterectomy [8]. On the other hand, such an extensive surgical approach may result in severe postoperative complications like lower leg lymphedema or vascular and nerve injury [9,10,11].

Lymphatic flow from the tumor goes to the first lymph nodes, called sentinel lymph nodes (SLN) where cancer cells are trapped and can be detected as metastases [12]. Over the last twenty years, different SLN mapping tracers have been tested in CC patients, mastering the SLN biopsy technique and gradually developing its efficacy and accuracy [7,9,13]. Finally, recent prospective studies have demonstrated the SLN technique combined with ultrastaging pathological processing significantly increased detection of low-volume lymphatic metastases, resulting in improved diagnosis of the early-stage CC patients with enhanced relapse risk but avoiding long-term morbidities, especially lower-leg lymphedema [4,5,14,15]. Consequently, questions have emerged of abandoning PLND for lymphatic mapping in favor of SLN biopsy alone; however, the oncological safety of such a novel approach must be confirmed in randomized prospective trials that are presently on-going [16,17].

The SLN lymphatic mapping effectiveness and sensitivity depend on a surgeon’s experience and a reliable tracer. Undoubtedly, most frequently used tracers (radioisotopes and dyes) for the SLN mapping in the early-stage CC patients have many advantages; however, they possess some flaws and limitations as well. Radiocolloids cannot visualize afferent lymphatic vessels and demand nuclear medicine facility and a special approach as they produce some radioactive exposure. In addition, they are quite expensive and associated with many logistic issues that could be troublesome. Dyes can be well visualized both in LNs and afferent lymphatic channels, thereby providing a more accurate SLN detection [18]; however, they diminish quite fast, and even small damage to lymphatic vessels during preparation may cause tracer leakage, which can impair SLN mapping. Additionally, the most popular dye tracer at the time of publication, indocyanine green (ICG), requires a high-tech, near-infrared imaging system to detect SLNs, which can be hardly affordable for many countries with low human development index, currently facing a growing CC pandemic [19].

Another optional SLN detection technique relies on administration of superparamagnetic iron oxide (SPIO) nanoparticles with mean hydrodynamic diameter of 60 nm, including carbodextran coating. These superparamagnetic molecules react to an external magnetic field induced by a magnetometer, giving a readable mark. After interstitial injection SPIO nanoparticles drain naturally from tumor via the lymphatic channels to the SLNs where they are concentrated and retained temporarily expressing superparamagnetic character reacting to an external magnetic signal but preserving no activity in the absence of SPIO [20,21,22]. Moreover, SLNs brownish colorization due to SPIO concentration acts as a visual dye that helps intra-operative identification. Hereby, the SPIO technique of SLNs detection can be both quantitative and qualitative [23].

Many prospective studies confirmed the feasibility of SLN mapping with the use of SPIO in breast cancer [20,21,24,25]. The effectiveness of a superparamagnetic nanoparticles in SLN mapping has also been reported in other malignancies such as prostate cancer [26], endometrial cancer [27], and vulvar cancer [28,29]. Nonetheless, it has hardly been studied in CC patients. So far, just one study demonstrated the use of SPIO as a tracer for SLN detection and identification of metastatic LN in uterine cancer patients (twelve cases with endometrial cancer and three with CC) in combination with preoperative magnetic resonance imaging (MRI) (but after SPIO administration) and LN iron staining [30].This pilot study aimed to investigate the diagnostic value and safety of SPIO nanoparticles of SPIO molecules administered intracervically for SLN mapping with a hand-held magnetometer in early-stage CC patients, submitted to open surgery and proceeded with PLND, following current recommendations [31,32]. The present study was a further development of our previous experience concerning SLN detection with a novel technique using a superparamagnetic tracer in patients with early-stage vulvar cancer and endometrial cancer [27,28], aimed at evaluating the efficacy of this method in gynecological cancers.

## 2. Materials and Methods

This observational and prospective trial was carried out in a single institution, Department of Gynecological Oncology at Wroclaw Medical University and Lower Silesian Oncology, Pulmonology, and Hematology Center in Wroclaw, Poland. The study project was endorsed by the Wroclaw Medical University Bioethics Committee (registration No: KB-780/2019, dated 2 December 2019), and the patients were enrolled between March 2020 and December 2023. This clinical investigation was conducted in accordance with the ethical principles that have their origin in the Declaration of Helsinki. All patients participating in this study gave a written informed consent. 

### 2.1. Study

Detailed information on sample acquisition, patient selection criteria, ethical approvals, and associated clinical data are provided in a previous publication [27]. In the present study, the gynecological tumor examined was characterized by a different anatomical location and clinicopathological features. The study population was entirely different from the previously studied group of patients. The women included in this study met the following conditions: diagnosis of primary CC (both squamous and adenocarcinoma), an apparent early stage according to FIGO classification (IA2 to IIA1), qualification by a multidisciplinary therapeutic team for surgical management of abdominal radical hysterectomy with or without bilateral adnexal resection (depending on patients’ age and tumor histological features) and SLN biopsy procedure followed by PLND as a standard of care (extended to the level of inferior mesenteric artery in case of high grade tumors and/or adenocarcinoma; no suspicion of LN metastases (N0) in clinical and radiological examination (MRI); distant metastases excluded preoperatively (M0); written informed consent of all participants in the study. We excluded from the study patients with suspicion of metastatic LNs and any other metastases; previous pelvic radiotherapy; any intolerance or allergy to iron oxide, or an iron cumulating disease; a metal implant in the pelvis; absence of written consent; intellectual disability; pregnancy or lactation in women of childbearing age.

The primary endpoint of the study was the proportion of patients with efficiently detected SLNs with the SPIO study method (detection rate), both overall and bilateral. The secondary endpoints were average SLNs per patient, percentage of SLNs detected from all dissected LNs (nodal detection rate), and the proportion of pathologically positive results (malignancy detection rate) per patient and per node. The proportion of patients with metastatic LNs including positive SLNs determined sensitivity of this study, negative predictive value (NPV) described the proportion of the patients with negative SLNs who had no metastatic LNs, and a false-negative rate (FNR represented the population of the patients with metastatic LNs who showed negative SLNs or failed to identify LN metastases with the study tracer. Any reported adverse event related to the SPIO SLN mapping technique, up to 30 days post-surgery, constituted the safety endpoints of the study.

### 2.2. Patients

From the total number of 32 patients in the study, 30 cases were included in the analysis. Two patients were excluded as their surgical procedure was performed with two days’ delay due to logistic issues that violated the study protocol. However, SLNs were detected in those patients with the magnetometer signal read proving a long-lasting effect of the SPIO cervical injection. The data of eligible patients were anonymized and recorded in the study case report form containing the following: demographics, surgical description, SLN procedure information, such as a time of cervical injection, SLN localization, a time of the SLN mapping, activity recordings (counts, colorations), and the pathological evaluation of SLNs and other specimens. The study investigators were required to assess and report any adverse events possibly related to the SPIO technique. The general characteristics of the study population are demonstrated in Table 1.

### 2.3. Surgical Procedure

In this study, we used the SPIO agent, a ready-made solution for the standard use as a tracer in soft tissue tumors (Magtrace^®^, Endomagnetics Ltd., Cambridge, UK). This blackish-brown sterile aqueous suspension of SPIO coated with carboxydextran, is a nanoparticle tracer that is intended and calibrated for use with a magnetic field detector (SentiMag^®^, Endomagnetics Ltd., Cambridge, UK). Both are CE-certified as class IIa medical devices. The carboxydextran coating SPIO prevents agglomeration while maintaining biocompatibility. The Z-averaged particle diameter, including the organic coating, is 60 nm (<0.25 polydispersity). Each milliliter of Magtrace^®^ contains approximately 28 mg of iron and is manufactured as a two mL aseptic and single-use vials, which is equivalent to a recommended dose of 55 mg of iron ±4 mg [33].

**Figure 1:**
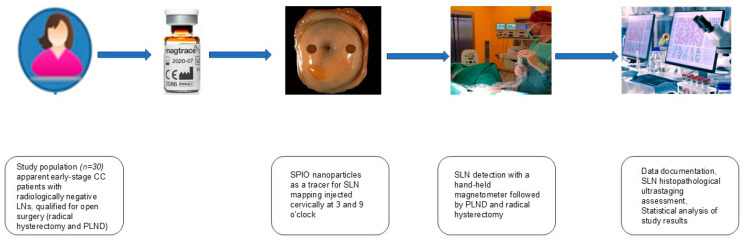
Schematic illustration of the study protocol of SLN mapping with SPIO nanoparticles as a tracer (Magtrace^®^, Endomagnetics Ltd., Cambridge, UK) and a hand-held magnetic detector (SentiMag^®^, Endomagnetics Ltd., Cambridge, UK) in early-stage CC patients. Abbreviations: CC—cervical cancer; SPIO—superparamagnetic iron oxide; LN—lymph node; SLN—sentinel lymph node; PLND—pelvic lymph nodes dissection. Figure adapted from [27].

On the eve of the planned surgery, a 2 mL vial of undiluted SPIO, was administered with a 23G needle under the cervical epithelium, superficially (about 1–3 mm) and deeply (about 10–15 mm) at 3 and 9 o’clock (1 mL each side, 0.5 mL for each superficial and deep injection). Then, an open abdominal surgery was accomplished by four different high-volume gynecological surgeons, who applied the same anatomic template for this procedure, with the assessment of abdominal and pelvic organs to rule out any macroscopic lesions followed by a retroperitoneal LN inspection along large vessels from obturator fossa bilaterally in the pelvis, till the origin of inferior mesenteric artery from the aorta. In order to avoid any interference with a hand-held, magnetic field detector, all metal instruments from LNs proximity were removed; instead, we used plastic retractors. LNs were thoroughly scanned with a handheld magnetometer to find the tracer signal. We placed the tip of the probe to LNs at very close range, as at such distance the measurement of LNs with SPIO uptake was demonstrated to be the most effective [34]. Based on our previous study experience with the SPIO technique [27,28], we considered every LN as an SLN if the magnetic count reading demonstrated at least a 5 times higher signal compared to a background LN count with the highest reading from the magnetometer probe and/or when LN brownish coloration was observed. Each excised SLN was assessed with a magnetometer ex vivo to confirm an in vivo signal read and documented (anatomical localization, signal values, brownish staining). All SLNs were then assessed with our institutional ultrastaging protocol by two experienced pathologists. Every metastatic LN was classified in accordance with the American Joint Committee on Cancer staging definition for axillary LN metastasis in breast cancer (macrometastases—tumor cells infiltration >2 mm, micrometastases < 2 mm, and isolated tumor cells <0.2 mm) [35]. After harvesting all SLNs the surgeons performed systemic PLND and a radical hysterectomy to accomplish the intentioned treatment plan. Every specimen was thoroughly examined with the use of our institutional pathology protocol for CC. Figure 1 illustrates the study design.

### 2.4. Statistical Analysis

The study data were computed and assessed as mean (±SD) and median (interquartile range (IQR)) with a statistical software (STATISTICA version 13.3, TIBCO Software Inc., Palo Alto, CA, USA). Detection rates were evaluated both at the patient and node level. SLN metastases revealed with SPIO nanoparticles and a magnetometer, indicated sensitivity and positive predictive value (PPV). The data obtained from PLND allowed us to calculate true negatives, specificity and NPV. The diagnostic efficiency of the SPIO detection experiment discriminating metastatic SLNs from metastatic LNs was estimated using the Receiver Operating Characteristic (ROC) curve with the area under the ROC curve (AUC) analysis, illustrating how the SPIO technique of SLN mapping distinguishes between these study cohorts.

## 3. Results

**Figure 2:**
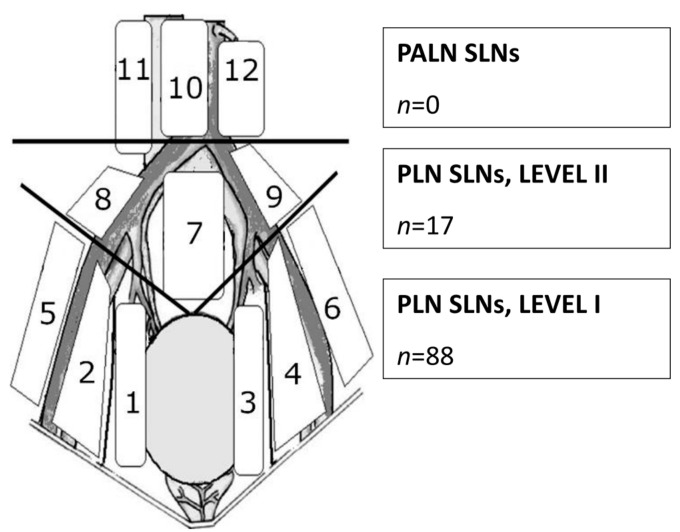
Anatomic localization of sentinel lymph nodes detected with a superparamagnetic iron oxide technique in early-stage cervical cancer patients. Abbreviations: PALN SLNs—para-aortic sentinel lymph nodes, PLN SLNs LEVEL I—pelvic sentinel lymph nodes beneath bifurcations of common iliac vessels, PLN SLNs LEVEL II—pelvic sentinel lymph nodes above bifurcations of common iliac vessels, 1—right obturator (*n* = 20), 2—right internal iliac (*n* = 17), 3—left obturator (*n* = 19), 4—left internal iliac (*n* = 20), 5—right external iliac (*n* = 5), 6—left external iliac (*n* = 7), 7—presacral (*n* = 1), 8—right common iliac (*n* = 8), 9—left common iliac (*n* = 8), 10—aortocaval (*n* = 0), 11—paracaval (*n* = 0), 12—para-aortic (*n* = 0).

SLNs were detected in every study patient (the overall detection rate was 100%); however, bilaterally in 27 patients (90%). There were 105 SLNs found in the entire study group. The proportion of SLNs detected per patient was, on average, 3.5 (range 1–8). All SLNs were located in the pelvis. For better clinical usefulness, we divided the pelvic lymphatic basin into two well-defined anatomical spaces, namely levels I and II [14]. The majority of pelvic SLNs (presented in Figure 2) were located in the lower region of PLN (from common iliac vessels bifurcations to obturator fossa vessels—level I), where we found 88 SLNs (83.8%). The rest of SLNs (*n* = 17, 16.2%) were detected in the upper part of PLN (from vena cava bifurcation to common iliac vessels bifurcations—level II).

Of note, 101 SLNs (96.2%) were described as black-brown (due to the SPIO tracer colorization) that visually aided the essential detection performed with a magnetometer use. Following the SLN detection procedure, we performed systematic PLND removing altogether 491 nodes, which resulted with average 16.3 lymph nodes dissected per patient (range 10–33). The mean SLN signal count was registered as 3148 (2991 ± SD), with a mean background reading after the SLN removal of 21 (11 ± SD). The median (IQR) SPIO detection signal count from all discovered SLNs was 2000 (650–5300) vs. a 20 (10–30) count read from PLND while the SPIO read from metastatic SLNs was clearly increased (median count 4700 with IQR 1490-7050). The mean time for the tracer distribution was 20 h (range 18–22 h) and did not affect the signal intensity read from the studied SLNs. The results of the SLN signal read are presented in Table 2.

Pathological analysis showed four patients with involved SLNs in the study cohort. Every of those cases had a single positive SLN (3 with micrometastases, diagnosed due toultrastaging protocol and 1 with macrometastases), but only in one patient there was additionally a positive LN (non-SLN) with macrometastases, located in the parametrium. There were no cases with positive LNs when SLNs were negative. Of note, in all positive SLN cases substantial LVSI was presented in the cervical tumor. Table 3 demonstrates study cases with metastatic LNs in the aspect of their anatomical localizations and tumor clinicopathological features.

**Figure 3:**
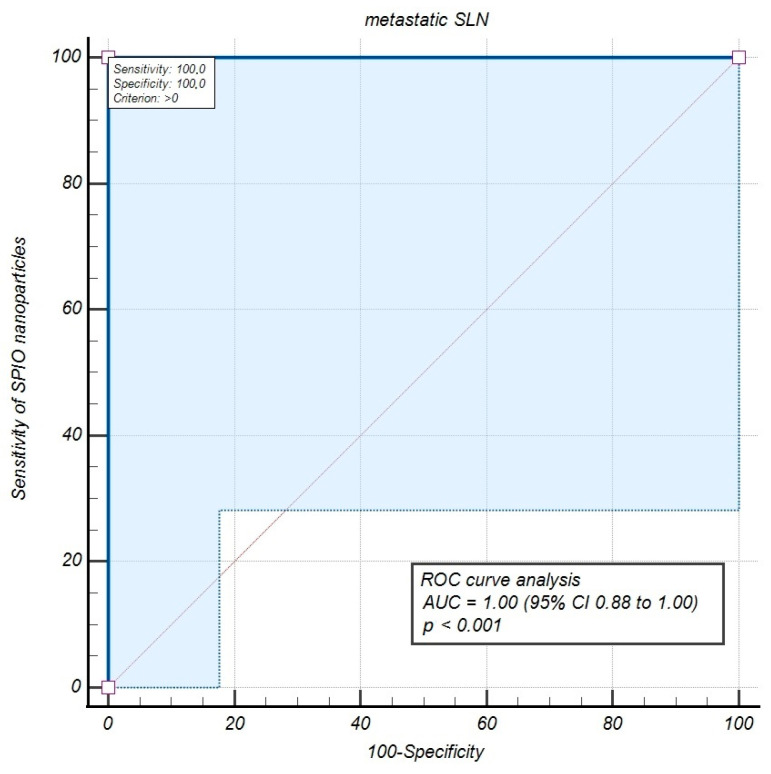
The efficacy parameters of the superparamagnetic iron oxide detection of metastatic sentinel lymph nodes evaluated by Receiver Operating Characteristic (ROC) curve analysis. Abbreviations: SPIO—superparamagnetic iron oxide; SLN—Sentinel Lymph Node; AUC—Area Under Curve.

The general malignancy rate for patients was 13.3% (4 of 30 patients had metastases in resected lymph nodes), while the malignancy rate for nodes was only 0.8% (5 positive nodes of all 596). We calculated the malignancy detection rate of SLNs per patient as 100% and per node as 80%. Therefore, the statistical analysis of our study data demonstrated a sensitivity of 100%, of the SLN metastases detection with the SPIO method compared with LND, as the study control. Consequently, specificity, PPV, and NPV were 100%, with an FNR of 0%. The diagnostic accuracy parameters of the SPIO detection of metastatic SLNs appraised by the ROC curve test with AUC, demonstrated a sensitivity of 100% (95% CI 39.8 to 100.0), and AUC of 1.00 (95% CI 0.88 to 1.00), corroborating the superparamagnetic molecules’ effectiveness in SLN mapping in women with early-stage CC (Figure 3).

Considering the safety of the study procedure no serious adverse events were reported.

## 4. Discussion

In this preliminary study we demonstrated our initial clinical experience with SLN detection technique with the use of superparamagnetic molecules administered cervically and mapped with a magnetic field detector in an early-stage carcinoma of the cervix. We were able to detect at least one SLN in all study cases while bilateral detection rate was presented in 90% of the study patients. The sensitivity and NPV were both 100%.These high SLN mapping accuracy parameters combined with pathologic ultrastaging assessment allowed us to diagnose low-volume disease in LNs that could be missed by standard pathologic processing after PLND. No adverse events related to the SPIO technique were reported.

For the first time feasibility and safety of the SPIO detection method for SLN mapping has been studied in early-stage CC patients, submitted to open surgery. The SPIO nanoparticles combined with a magnetometer use seem to be an effective method for SLNs detection in this malignancy, as we presented the overall detection rate of 100%, with bilateral rate of 90%; however, direct comparative studies to other tracers and detection techniques have not been conducted so far thus, the feasibility of SPIO use in SLN mapping should be considered carefully. In earlier studies, blue dye or radioisotope injection (technetium-99) were used as tracers alone or in combination which demonstrated the detection rate of 99.1% in comparison with 92.8% of blue dye as a single marker [36]. Regardless of the used tracer or tracers, both overall detection rate and sensitivity were reported as 92.3% in the large meta-analysis for SLN mapping in CC patients, which was assessed as acceptable [37]. Recently, near-infrared fluorescence (NIR) imaging with ICG has dominated the SLN mapping in gynecological cancers mostly due to a technological breakthrough with surgical robotic platforms implementation what resulted in the detection rate progression from 88% to 98.5% due to growing surgeons’ experience [18,38]. Unlike a radiotracer or SPIO nanoparticles, an ICG with a dedicated endoscopic NIR system is able to visualize the afferent lymphatic channels pathways: upper paracervical (UPP) and lower paracervical (LPP), facilitating SLN mapping due to the anatomical lymphatic drainage of the uterus [39]. Therefore, it has been recently recommended as a surgical guide for SLN mapping in CC by an international panel of expert, gynecological oncology surgeons [40]. Still, in a case of open surgery when visualization of pelvic lymphatic vessels is hardly possible, the SPIO detection technique may be potentially useful if validated comparatively with other tracers.

The bilateral detection rate and sensitivity are crucial parameters to assess effectiveness of the SLN idea and can be evaluated as trustworthy when such a proceeding is ensued by systemic LND as the control. In order to evaluate the feasibility of the SPIO technique as an alternative SLN mapping in early-stage CC patients, we confronted our data with many large prospective and retrospective studies utilizing other detection technologies, since the literature regarding superparamagnetic mapping of SLNs in this cancer is lacking. In the SENTICOL study, the authors reported a sensitivity of 92%, NPV of 98%, 97.8% of overall detection rate, and no false-negative results in 76.5% of patients with the bilateral detection [41]. The comparison with further SENTICOL II study demonstrated that surgical experience is the main factor for the increase in SLN successful bilateral detection [42]. According to this study, other clinical features, such as tumors larger > 20 mm, BMI > 30 kg/m^2^ and age > 70 years, may cause the failure of bilateral SLN mapping. Another prospective study by Lührs et al. showed an ICG-based SLN detection algorithm with a strict SLN definition resulting in sensitivity and NPV of 100% [39]. Thorough assessment of the anatomical lymphatic drainage from the uterus demonstrated bilateral mapping rate of UPP at level of 97.2% and LPP of 69.7%. The authors concluded that pelvic SLN anatomical distribution is similar both in cervical and endometrial carcinoma. The SLN detection based on a strict anatomical algorithm is a safe alternative to PLND in early-stage CC patients [40]. Undoubtedly, the prerequisite for the successful, high-rate bilateral SLN mapping in cervical carcinoma is systematic, following the anatomical lymphatic drainage from the parametria. The largest prospective trial so far, SENTIX, showed the overall SLNs bilateral detection rate in 91% of the study cases (with different tracers used), which was unaffected by tumor size, tumor stage, LVSI, or BMI but was significantly lower in older patients (>60 years) and low-volume cases study centers [14]. Our study demonstrated 90% bilateral detection rate and similarly no clinical factors (tumor stage, size, stromal infiltration, LVSI, BMI, age) influenced this parameter in our observation. Due to this high bilateral SLN detection followed by pathological ultrastaging assessment we could report 100% sensitivity and NPV, with no FNR. These results correspond not only to the prospective studies mentioned above (Table 4) but also to large retrospective investigations, with 97% sensitivity in bilateral SLN detection subgroup (false-negative rate was 2.8%) and with a sensitivity of 96.4% and NPV of 99.3% (false-negative rate was 3.6%) [7,41,43].

Likewise, the results of a large meta-analysis (44 studies encompassing 3931 cases) were similar with a pooled sensitivity of 94%, NPV ranging between 91 and 100%, and false-negative rate of 0.08% if prerequisites, such as tumor size < 40 mm and bilateral negative SLNs after ultrastaging processing, were present. Pooled data on a combined tracer with cervical tumor < 20 mm resulted in a87% bilateral SLN detection rate [44].

The median number of detected SLNs per patient was 3.5 in our study, whichis in-line with the SENTIX trial (3 nodes per patient) [14]. Similarly to this large prospective study, we report the SLNs localization with majority of them found in level I of the pelvis (83.8% vs. 90% of SLNs in the SENTIX trial) and the rest in level II region. Identically, we could not detect any atypically located SLNs or para-aortic SLNs. We found all metastatic LNs in the pelvic level I. This observation was supported by the SENTIX study results, with only 2% of isolated metastatic SLNs in level II and 98% of involved SLNs in level I. The authors concluded that skip-metastases to para-aortic LNs were very unlikely in early-stage CC and that surgeons should pay particular attention to the pelvic region below common iliac vessels bifurcation [14]. Of note, we found three cases (of four with nodal disease) with metastatic SLNs but without additional nodal involvement (non-SLNs). However, one case had both positive SLN and an isolated LN (with macrometastasis) in the parametrium. It is not uncommon to find metastatic LNs in parametria, as they have typical drainage from the cervix and therefore, the parametria are usually resected during radical hysterectomy, being an integral part of surgical specimen. Still, the reliable detection of SLNs in the parametria remains difficult or even impossible due to a tracer leakage to the adjacent tissue impairing the signals and/or lymphatic channels and nodes colorization [45].

The weaknesses of our investigation include a single institution experience and a low-volume study population that certainly is underpowered to definitely demonstrate the diagnostic value of the SPIO nanoparticles for SLN detection in early-stage CC patients. However, the research project of our pilot study, including a small investigated group, resulted from the original presupposition that the SPIO technique could be valuable for SLN detection in early-stage CC patients submitted to open surgery, without further distinctions. As a result of the decreasing number of early-stage CC patients, the accumulation of early-stage CC cases and endometrial cancer patients could facilitate a methodologically sufficient and adequate powered SLN studies on sensitivity in cervical carcinoma [39]. Based on our initial clinical experience from this study, we regard the SPIO procedure as potentially efficient in the SLN mapping of apparent early-stage CC patients, particularly in open surgery, which is, however, broadly accomplished for radical hysterectomy as a consequence of the Laparoscopic Approach to Cervical Cancer (LACC) study results [46]. The SPIO detection procedure for SLN mapping is easy to handle in comparison with radiotracers and/or dyes used during open surgery, and the learning curve is rather short, especially in the institutions that are experienced with its application for SLN detection in other malignancies. The SPIO tracer collection in SLNs produces visible and audible signal, assisted with SLN coloring. Its use is safe for patients as we did not report any adverse events associated with this novel tracer. Avoiding the exposure to ionizing radiation in the SPIO technique makes the safety of the patients and staff undisputable in comparison with the standard use of a radiocolloid tracer. Last but not least, in our country, the SPIO procedure is significantly more cost-efficient than the standard radioisotope technique or NIR imaging systems for minimal invasive surgery using ICG as a tracer for SLN mapping.

## 5. Conclusions

The present preliminary study indicates that the SLN detection technique employing superparamagnetic nanoparticles and a hand-held magnetometer could become a safe and effective alternative to PLND in early-stage cervical carcinoma patients managed with open surgery. The diagnostic value of the SPIO detection of metastatic SLNs, evaluated by ROC curve analysis with the area under the ROC curve (AUC), revealed a sensitivity of 100% and an AUC of 1.0 (*p* < 0.001), confirming SLN mapping with SPIO to be significantly effective in women with early-stage CC. However, this initial clinical experience includes a small study group, requiring further research to validate these data, especially through comparison with other SLN mapping techniques.

## Figures and Tables

**Table 1:** General characteristics of the study population (*n* = 30).

Age (years) (mean (range))	46.9 (31–76)
BMI (kg/m^2^) (mean (range))	25.1 (17.3–40.9)
Squamous cancer (*n*)	26
Adenocarcinoma (*n*)	4
Tumor size (mm) (mean (range))	18.0 (3–45)
Interstitial cervical infiltration (mm) (mean (range))	7.5 (1–25)
Stage pT1A2 (*n*)	2
Stage pT1B1 (*n*)	16
Stage pT1B2 (*n*)	7
Stage pT1B3 (*n*)	4
Stage pT2A1 (*n*)	1
Low grade tumor (*n*)	26
High grade tumor (*n*)	4
LVSI positive (*n*)	9
LVSI negative (*n*)	21

Abbreviations: BMI—body mass index, LVSI—lymphovascular space involvement.

**Table 2:** Superparamagnetic iron oxide detection signal readings in dissected lymph nodes from women with cervical cancer. Study results.

Statistical Analysis	SLN_ALL Count	SLN_OBT Count	SLN_INT_ILIAC Count	SLN_EXT_ILIAC Count	SLN_COM_ILIAC Count	SLN_MET Count	LND Count
Mean	3148	3513	2666	2244	3212	4270	21
SD	2991	3019	2972	2112	3089	3316	11
Median	2000	2500	1398	1600	2000	4700	20
25% centile	650	650	370	680	650	1490	10
75% centile	5300	5800	4500	3500	6100	7050	30
IQR	4735	5150	4373	2820	5450	5560	20

Abbreviations: SLN_ALL—all dissected sentinel lymph nodes, SLN_OBT—obturator sentinel lymph nodes, SLN_INT_ILIAC—internal iliac sentinel lymph nodes, SLN_EXT_ILIAC—external iliac sentinel lymph nodes, SLN_COM_ILIAC—common iliac sentinel lymph nodes, SLN_MET—metastatic sentinel lymph nodes, LND—all dissected lymph nodes from systematic lymphadenectomy, SD—standard deviation, IQR—interquartile range.

**Table 3:** Study patients with metastatic lymph nodes. Clinicopathological characteristics.

Clinical Features	SLN	LND	Tumor Type	Tumor Size (mm)	Interstitial Infiltration Depth (mm)	Grade	LVSI
**CASE 1**							
Nodal metastasis detection method	*n* = 1	*n* = 0					
Metastatic LN localization	right obturator						
Metastasis type	MIC						
Pathological assessment			squamous	33	15	LG	positive
**CASE 2**							
Nodal metastasis detection method	*n* = 1	*n* = 0					
Metastatic LN localization	right obturator						
Metastasis type	MIC						
Pathological assessment			adenocarcinoma	10	3	LG	positive
**CASE 3**							
Nodal metastasis detection method	*n* = 1	*n* = 0					
Metastatic LN localization	right internal iliac						
Metastasis type	MAC						
Pathological assessment			squamous	20	10	LG	positive
**CASE 4**							
Nodal metastasis detection method	*n* = 1	*n* = 1					
Metastatic LN localization	left obturator	left parametrium					
Metastasis type	MIC	MAC					
Pathological assessment			squamous	10	6	LG	positive

Abbreviations: LN—lymph node; SLN—sentinel lymph node; LND—lymphanedectomy; LG—low grade; LVSI—lymphovascular space involvement; MIC—micrometastasis; MAC—macrometastasis.

**Table 4:** Comparison between prospective studies on the accuracy of sentinel lymph node detection with different tracers in early-stage cervical cancer patients and the results of the present study using superparamagnetic iron oxide nanoparticles (SPIO study).

Study	Patients (*n*)	Tracer	Sensitivity (%)	Bilateral Detection Rate (%)	Negative Predictive Value (%)	False Negative Rate (%)
SENTICOL [41]	136	Tc^99^+BD	92.0	76.5	98.2	1.5
SENTIX [14]	395	Tc^99^+BD; BD; ICG	N/A	91.0	N/A	N/A
Lührs et al. [18]	65	ICG vs. Tc^99^	N/A	98.5(ICG) vs. 60(Tc^99^)	N/A	N/A
Lührs et al. [39]	145	ICG	100.0	97.9	100.0	0.0
SPIO	30	SPIO	100.0	90.0	100.0	0.0

Abbreviations: SPIO—superparamagnetic iron oxide; ICG—indocyanine green; Tc^99^—radioisotope technetium 99; BD—blue dye; N/A—no available.

## Data Availability

The raw data supporting the conclusions of this article will be made available by the authors on request.
